# Acquired platelet dysfunction in newly diagnosed myeloma

**DOI:** 10.1002/jha2.152

**Published:** 2021-01-15

**Authors:** Joshua Mortimer, Nicola Gray, Michael Desborough, Peter Baker, Jaimal Kothari, Toby Eyre

**Affiliations:** ^1^ Department of Haematology Oxford University Hospitals NHS Foundation Trust Oxford UK; ^2^ Oxford Haemophilia and Thrombosis Centre, Churchill Hospital Oxford UK

**Keywords:** bleeding disorders, multiple myeloma, platelet aggregation

## Abstract

This is a case of an unexpected and dramatic bleeding complication in a patient post‐bone marrow biopsy performed for investigation of an IgA paraprotein with the results confirming multiple myeloma. Subsequent investigations were suggestive of an acquired platelet disorder indicated by abnormal platelet light aggregometry readings. It was observed that the impaired platelet aggregation corrected on reduction of the paraprotein load following commencement of antimyeloma treatment and plasma exchange. This case is of significant clinical relevance as it highlights a risk factor for serious complication in patients undergoing procedures in those with untreated plasma cell dyscrasias.

A 66‐year‐old male with newly diagnosed IgA kappa multiple myeloma (International Staging System stage 3 with intermediate risk cytogenetics) developed swelling and pain at biopsy puncture site on day (D) 9 following an uneventful bone marrow aspirate and trephine biopsy (BMAT). A full blood count showed a haemoglobin of 88 g/L (normal range, 130‐170 g/L) ‐ a reduction from 118 g/L pre‐BMAT ‐ and a platelet count of 108 × 10^9^/L (normal range, 150‐400 × 10^9^/L). The activated partial thromboplastin time was 31.2 s (range, 20‐30) and prothrombin time 10.8 s (range, 9‐12 s) (Table 1). At this time, the total IgA level was 33 g/L with an associated low IgG and IgM. An ultrasound scan showed no organised collection or haematoma and he was discharged. On D15 post‐BMAT his symptoms worsened (Figures 1 and 2). His haemoglobin was 63 g/L and a CT angiogram revealed a large right buttock haematoma measuring 12 × 5.8 × 9.9 cm but no active extravasation. Three units of packed red blood cells were transfused. His factor VIII, IX von Willebrand levels and activity were normal.

**TABLE 1:** Blood test results

	Normal range	Day 11	Day 15	Day 36	Day 38	Day 44	Day 65
Prothrombin time	9‐12 s	10.4	10.4	10.5	NA	10	10.1
Activated partial thromboplastin time	20‐30 s	26.1	31	34.5	NA	27.1	24.9
Platelet count	150‐400 × 10^9^/L	104	120	123	104	65	240
Von Willebrand factor antigen	0.5‐2.0 IU/mL	NA	3.77	3.97	NA	2.91	NA
Factor VIII level chromogenic	0.5‐2.0 IU/mL	NA	4.79	4.79	NA	3.19	NA
Factor IX level	0.5‐2.0 IU/mL	NA	1.13	1.18	NA	1.07	NA
Factor XI level	0.7‐1.3 IU/mL	NA	0.59	0.42	NA	0.79	NA
Von Willebrand factor activity (GP1bR)	0.5‐2.0 IU/mL	NA	3.77	4.7	NA	3.6	NA
Von Willebrand factor collagen binding activity	0.5‐2.0 IU/mL	NA	3.66	NA	NA	2.87	NA
PFA‐200 collagen/epinephrine closure time	79‐164 s	NA	NA	NA	104	212	94
PFA‐200 collagen/adenosine diphosphate closure time	55‐112 s	NA	NA	NA	>300	>141	87

**FIGURE 1:**
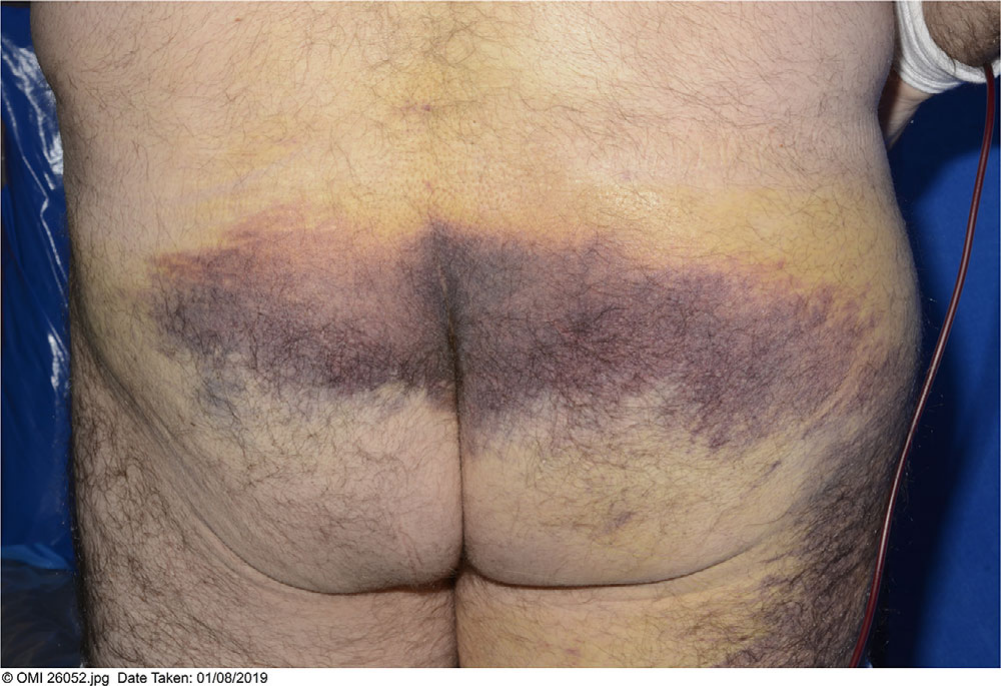
Presentation day 15 post bone marrow aspirate and trephine biopsy

**FIGURE 2:**
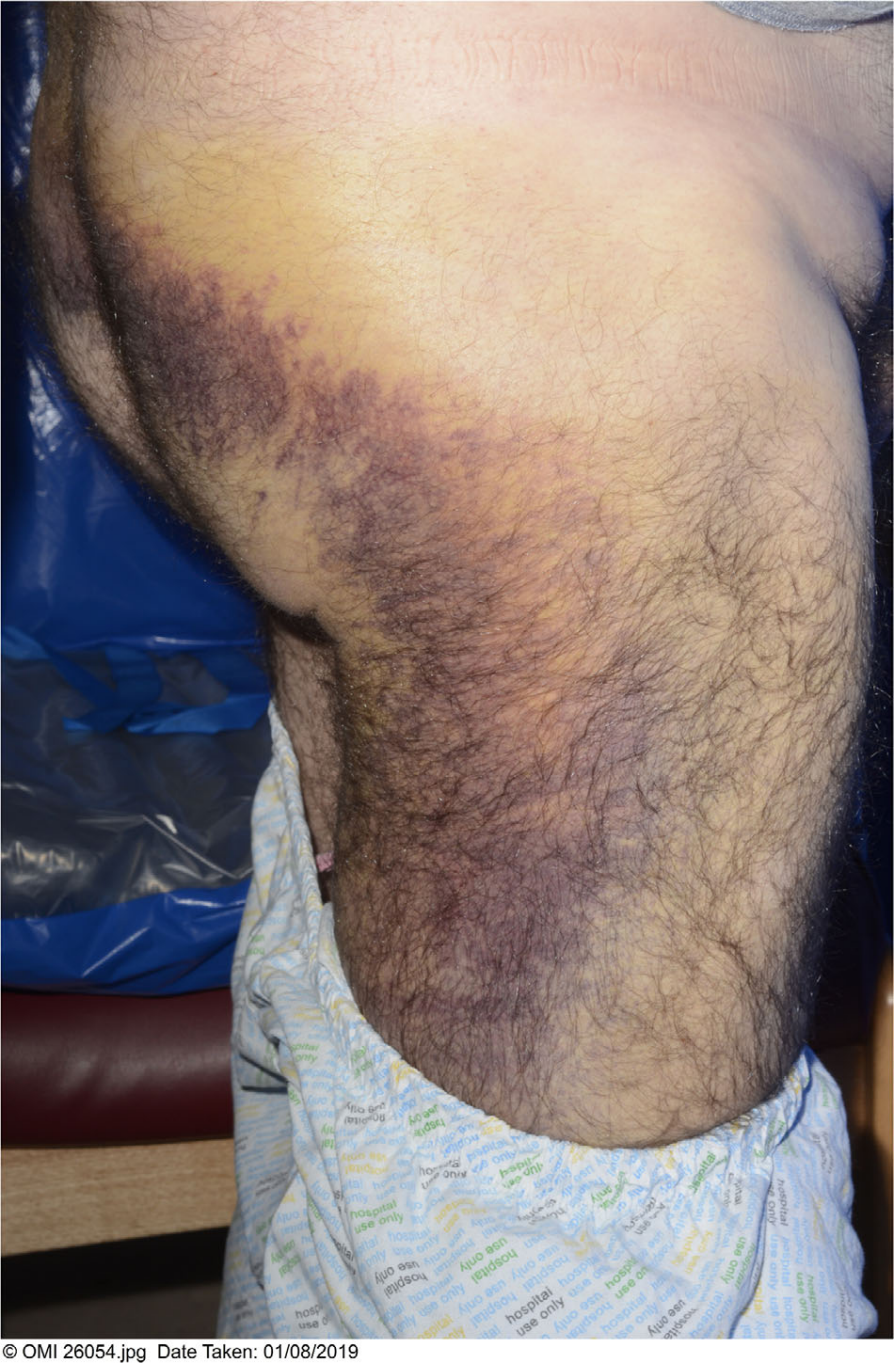
Presentation day 15 post bone marrow aspirate and trephine biopsy

On D17, he was initiated on bortezomib, cyclophosphamide and dexamethasone. On D34, he re‐presented with further bruising and a haemoglobin 67 g/L. Screening for platelet function using PFA‐200 closure time reported an impaired response to collagen/ADP (>300 s) (normal range, 55‐112) and collagen/epinephrine (212 s) (normal range, 79‐164). Subsequent light transmission aggregometry displayed impaired aggregation in response to collagen, ADP and adrenaline, further suggesting reduced platelet function (Figure 3). Settings were modified to account for the thrombocytopaenia and suggested additional interference of platelet function likely due to the underlying paraprotein.

**FIGURE 3:**
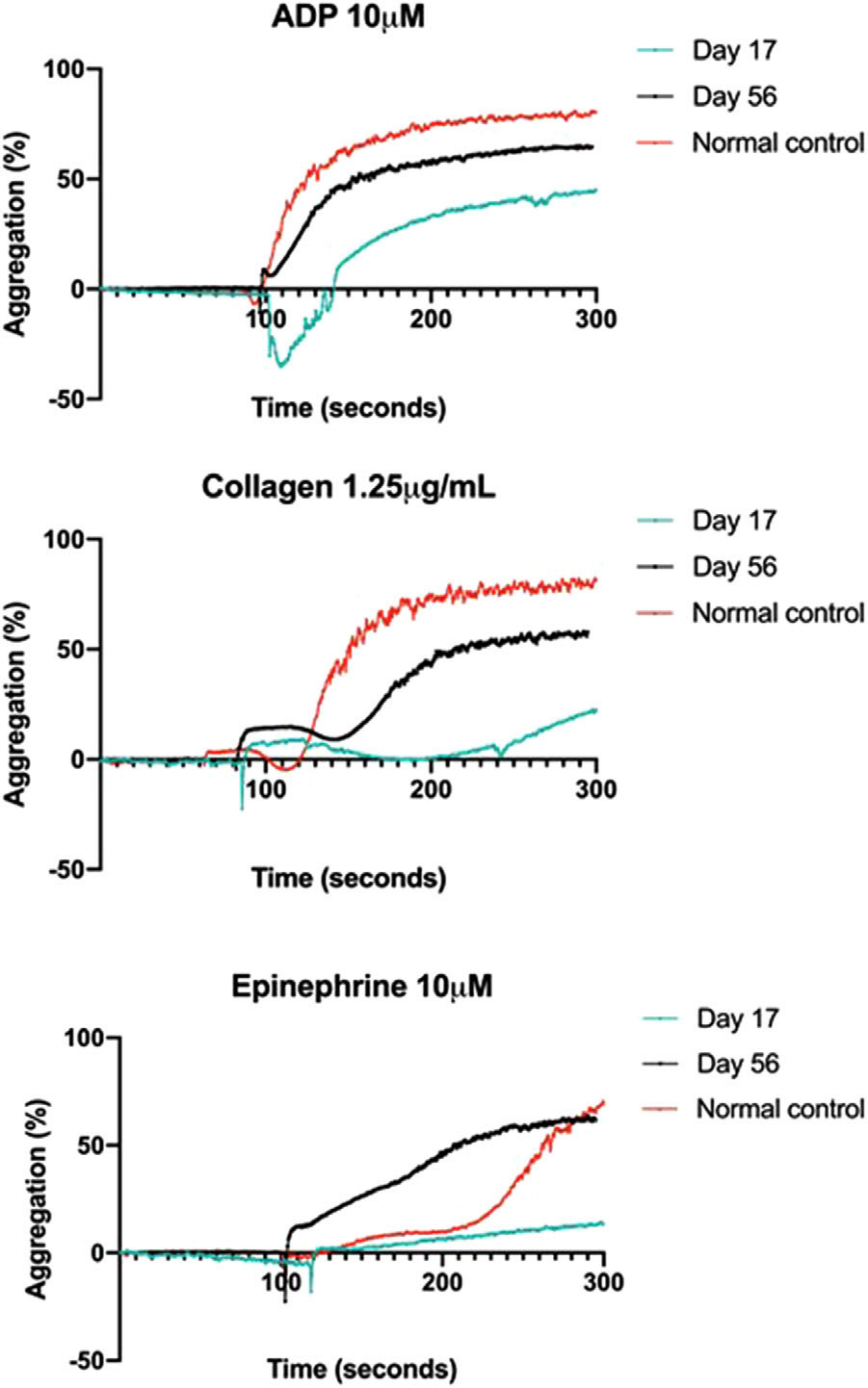
Platelet light transmission aggregometry curves

Plasma exchange (PEX) was instigated on D37 for a suspected paraprotein‐mediated platelet defect. His IgA fell to 16 g/L from 33 g/L after the initial PEX and he was discharged home on D43 with a third PEX on D49. Further platelet light aggregometry was performed on D56 when his platelet count was normal and paraprotein was 11 g/L (Figure 3). PFA‐200 closure time revealed normalisation of response to collagen/Epinephrine and collagen/ADP.

The dramatic and unusual bleeding pattern in the absence of a bleeding history and in association with a large IgA paraprotein was highly suggestive of an acquired, reversible paraprotein‐related platelet‐associated bleeding phenomenon. Paraproteinaemias are rarely associated with severe platelet dysfunction.[1] We demonstrate that paraprotein‐associated platelet dysfunction can result in clinically significant bleeding complications. Acquired platelet dysfunction must be considered in patients undergoing procedures or presenting with bleeding in those with untreated plasma cell dyscrasias.
